# Systemic inflammatory response index mediates the association between stroke and hypertension: a cross-sectional study from NHANES 2001 to 2016

**DOI:** 10.3389/fneur.2025.1603241

**Published:** 2025-07-30

**Authors:** Mingzhu Tang, Fangming Diao, Yaxian Dong, Jianpeng Lin, Tiezhu Wang, Lihua Yang, Xuanming Lai, Xiaolian Chen, Jinxi Zuo, Junyang Xu, Hongting Shi

**Affiliations:** Department of Neurology, Institute of Neuroscience, Key Laboratory of Neurogenetics and Channelopathies of Guangdong Province and the Ministry of Education of China, The Second Affiliated Hospital, Guangzhou Medical University, Guangzhou, China

**Keywords:** stroke, hypertension, systemic immune response index, inflammation, cross-sectional study, mediation

## Abstract

**Background:**

Systemic inflammation plays a vital role in the development of hypertension and stroke. The systemic immune response index (SIRI), calculated from the numbers of neutrophils, monocytes, and lymphocytes, is a promising indicator of immune dysregulation. Yet, its role in mediating the link between hypertension and stroke remains underexplored.

**Methods:**

This study analysed data from 9,699 adults aged ≥20 years from the National Health and Nutrition Examination Survey (NHANES) 2001–2016. We used logistics regression analyses, Receiver Operating Characteristic (ROC) curve analyses, mediation analyses, trend tests, restricted cubic splines (RCS) and stratified by sex, to explore the associations between SIRI, hypertension, and stroke.

**Results:**

SIRI was significantly associated with stroke (adjusted OR = 1.10, 95% CI: 1.00–1.21, *p* = 0.047). Hypertension was independently linked to both stroke (adjusted OR = 2.06, 95% CI: 1.42–2.99, *p* < 0.001) and higher SIRI levels (adjusted *β* = 1.07, 95% CI: 1.03–1.12, *p* = 0.002). ROC analysis confirmed strong predictive power for hypertension and SIRI in stroke risk assessment. RCS analysis revealed a nonlinear U-shaped relationship between SIRI and stroke in the overall population and males, but a flatter trend in females. SIRI mediated 1.65% of the hypertension-stroke association (*p* < 2 × 10^−16^), with a stronger effect in males (3.38%) than females (1.16%).

**Conclusion:**

Hypertension, SIRI, and stroke were closely related, with SIRI partially mediating their association, particularly in males. SIRI might be potential as a biomarker and therapeutic target for stroke prevention in hypertensive individuals.

## 1 Introduction

According to the most recent data by the Global Burden of Disease (GBD) 2021 study, stroke is an major global health issue, causing 160.4 million disability-adjusted life years (DALYs) in 2021 and ranking as the fourth leading cause of DALYs worldwide (1). It underlines its substantial burden of mortality as well as disability. Stroke-related risk factors permeate different dimensions like exposures to the environment, dietary habits, lifestyle orientations, and physical conditions (2). Notably, hypertension, characterized by elevated systolic blood pressure, is widely recognized as a primary risk factor for stroke, with its role particularly pronounced in spontaneous intracerebral hemorrhage and specific ischemic stroke subtypes, such as lacunar and atherothrombotic infarctions (3). Research indicates that hypertension is a major cardiovascular risk factor for these ischemic subtypes, which are associated with small and large artery diseases, respectively (4). In 2019, hypertension accounted for 55.5% of all disability-adjusted life years (DALYs) attributed to stroke (2). Hypertension elevates the risk of stroke through multiple mechanisms including vascular endothelial dysfunction, vascular remodeling, oxidative stress, immune dysregulation, and inflammation (5, 6). Of these, endothelial dysfunction and chronic systemic inflammation are postulated to be in a mutually reinforcing triad with hypertension. Endothelial dysfunction has been widely acknowledged as an early precursor of hypertension and is significantly modulated by chronic inflammation. This intricate interplay not only exacerbates microvascular and macrovascular complications but also heightens the risk of stroke in hypertensive individuals (7). Therefore, given the shared pathophysiological mechanisms between hypertension and stroke, identifying potential inflammatory markers may be of critical importance for predicting, screening, and preventing strokes in hypertensive populations.

In recent years, the systemic inflammatory response index (SIRI) has emerged as a novel inflammatory biomarker (8), garnering significant attention for its crucial role in hypertension and stroke research. Multiple studies have demonstrated that SIRI is significantly associated with all-cause and cardiovascular mortality in hypertensive patients (9, 10). Additionally, SIRI is associated with stroke severity, complications, and all-cause mortality (11). Its prognostic value extends to other cardiovascular events, such as non-ST-elevation myocardial infarction (NSTEMI) and intracerebral hemorrhage (12, 13).

In contrast, traditional inflammatory markers like high-sensitivity C-reactive protein (hs-CRP) exhibit limited sensitivity and specificity in comprehensively assessing and predicting stroke risk (14). Zhang at al. (11) found that SIRI had greater predictive value than hs-CRP. Meanwhile, A study using the National Health and Nutrition Examination Survey (NHANES) data further indicated that the after adjusting for confounders, SIRI was significantly associated with stroke history, whereas hs-CRP did not show a similar association (15). This suggests that SIRI may have a unique advantage in reflecting chronic inflammatory states associated with stroke, particularly in cross-sectional studies. As a biomarker derived from peripheral blood neutrophil, monocyte, and lymphocyte counts, SIRI can simultaneously reflect systemic inflammation and immune imbalance (8). Although non-inflammatory factors such as coronary heart disease, cancer may affect SIRI levels (16, 17), careful adjustment for confounding variables ensures that its association with stroke primarily stems from inflammatory processes.

Drawing on these studies (10–13), we hypothesized that SIRI may play a critical role in the relationship between hypertension and stroke, however, its specific role has not been elucidated. Therefore, leveraging data from the NHANES database, we investigated the association between hypertension and stroke and further examined the mediating effect of SIRI on this relationship. Moreover, prior studies have demonstrated that sex modifies the associations between inflammation, hypertension, and stroke (18–21). Given the existence of sex differences in the interplay among inflammation, hypertension, and stroke, we conducted stratified analyses to examine these associations separately in males and females.

## 2 Materials and methods

### 2.1 Study participants

NHANES is a cross-sectional study conducted by the National Center for Health Statistics (NCHS), an organization under the Centers for Disease Control and Prevention (CDC). This survey estimates the health and nutritional status of the civilian, non-institutionalized U. S. population using stratified, multistage probability sampling methods. Data collection employs standardized processes, including interviews, physical examinations, laboratory measurements, and dietary evaluation. NHANES data are available to the public and can be downloaded from: https://wwwn.cdc.gov/nchs/nhanes/default.aspx. The study has received approval from the NCHS Research Ethics Review Board, and all participants provided written informed consent.

**Figure 1:**
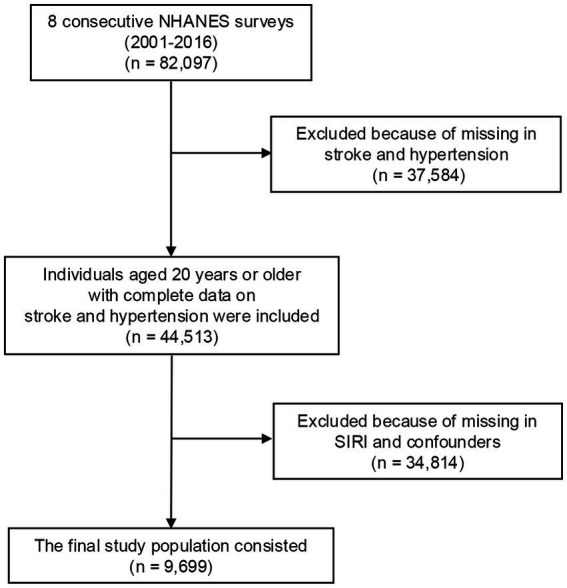
Flow chart of patient selection. NHANES, National Health and Nutrition Examination Survey; SIRI, systemic inflammation response index.

For our study, eight cycles of NHANES data (2001–2016) were pooled, with an original sample size of 82,097 participants. Participants were excluded if they had missing data on the following key variables: SIRI, hypertension, stroke status, demographic factors [e.g., marital status, education attainment, body mass index (BMI), poverty-income ratio (PIR)], lifestyle factors (e.g., smoking, alcohol consumption), and clinical conditions (e.g., coronary heart disease, dyslipidemia, cancer). These exclusions resulted in the removal of 72,398 individuals. As indicated in Figure 1, the final analytical sample included 9,699 participants.

### 2.2 Stroke

The outcome variable, stroke diagnosis, was primarily derived from self-reported medical histories collected as part of the NHANES survey. Participants were specifically queried regarding whether they had ever been diagnosed with stroke by a physician or other healthcare professional. Based on their responses, participants were classified into two groups: “stroke” and “non-stroke” (22).

### 2.3 Hypertension

Hypertension served as the exposure variable and was assessed using both blood pressure measurements and self-reported data. Blood pressure readings were obtained using a mercury sphygmomanometer after participants had rested in a seated position for at least 5 min. An appropriately sized cuff was securely wrapped around the right arm, and three consecutive measurements were recorded. In cases where any measurement was interrupted or deemed invalid, an additional fourth reading was performed. The mean values of systolic blood pressure (SBP) and diastolic blood pressure (DBP) were subsequently calculated.

Hypertension was defined according to the 2017 AHA/ACC guidelines, with criteria including SBP ≥ 130 mmHg and/or DBP ≥ 80 mmHg. Participants were also classified as hypertensive if they responded affirmatively to either of the following questions: “Have you ever been informed by a healthcare professional that you have hypertension?” or “Are you currently taking antihypertensive medication?” (23–25).

### 2.4 Systemic inflammation response index (SIRI)

SIRI is a novel composite indicator of inflammatory response and served as the mediating variable in our study. Peripheral blood samples collected from NHANES participants were analyzed at mobile examination centers (MEC) using a Beckman Coulter HMX hematology analyzer. Lymphocyte, neutrophil, and monocyte counts were quantified via complete blood count (CBC) and expressed in units of ×10^3^ cells/μL. The SIRI was subsequently calculated using the formula: (monocyte count × neutrophil count)/lymphocyte count (26, 27).

### 2.5 Covariates

In this study, we adjusted for potential confounding factors, encompassing demographic, lifestyle, and clinical variables. Demographic factors included age (categorized as 20–39 years, 40–59 years, and ≥ 60 years), sex, race/ethnicity (Mexican American, Non-Hispanic White, Non-Hispanic Black, Other Hispanic, and Other/Multiracial), marital status (married/living with a partner vs. widowed/divorced/separated/never married), education attainment (less than high school, high school graduate, or more than high school), and poverty-income ratio (PIR: categorized as ≤ 1, 1 to ≤ 3, and > 3). Lifestyle factors comprised smoking status (≥ 100 cigarettes in a lifetime or < 100 cigarettes) and alcohol consumption (≥ 12 drinks in a lifetime or < 12 drinks). Clinical factors included self-reported diagnoses of coronary heart disease and cancer by a healthcare professional, diabetes (defined as a prior diagnosis by a healthcare professional, current use of antidiabetic medication or insulin, or glycated hemoglobin [HbA1c] ≥ 6.5%) (28), and dyslipidemia. Dyslipidemia was also characterized based on whether any single or combination of the following levels had been reached: low-density lipoprotein (LDL) ≥ 3.4 mmol/L (≥ 130 mg/dL), total cholesterol (TC) ≥ 5.2 mmol/L (≥ 200 mg/dL), triglycerides (TG) ≥ 1.7 mmol/L (≥ 150 mg/dL), or high-density lipoprotein (HDL) ≤ 1.0 mmol/L (< 40 mg/dL). Although guidelines, such as those from the AHA/ACC, recommend sex-specific HDL cut points (men < 1.0 mmol/L and women < 1.3 mmol/L), a single cutoff for HDL (≤ 1.0 mmol/L) was used in this analysis to allow easier analysis and comparability across subgroups. This approach is in line with standard practice in big epidemiological studies to facilitate data comparison and processing (29). Body mass index (BMI) was categorized according to WHO standards as < 25 kg/m^2^, 25–30 kg/m^2^, and ≥ 30 kg/m^2^ (30).

### 2.6 Statistical analysis

The analyses were conducted per NHANES standards, considering the complex survey design elements, including sample weights, clustering, and stratification in order to supply nationally representative data. Statistical processing was performed via R software 4.5.0 under CDC guidelines. A two-tailed *p*-value of less than 0.05 was taken as a standard for statistical significance. Continuous measures were expressed in descriptive statistics in the form of median (quartile range: Q25%, Q75%). Categorical variables were expressed as frequencies (percentages). For comparisons of continuous variables between groups, the Wilcoxon rank-sum test, adapted to complex survey samples, was applied. For categorical variables, Rao and Scott’s second-order adjusted chi-squared test was applied.

First, univariate analyses were performed to screen for variables that might be associated with stroke. Variables were included in the multivariate logistic regression model if they were significant (*p* < 0.05) or were recognized as important confounders based on prior literature. Then, multivariate logistic regression analyses were conducted to examine the associations between hypertension and stroke, as well as SIRI and stroke. Multivariate linear regression analyses were performed to investigate the relationships between SIRI and hypertension. Model 1 included no adjustments for covariates. Model 2 adjusted for demographic variables, including age, sex, marital status, race/ethnicity, education attainment, and PIR. Model 3 was further adjusted for clinical variables, such as BMI, smoking status, alcohol consumption, dyslipidemia, coronary heart disease, diabetes, and cancer history.

After adjusting for all potential confounding factors, trend tests were conducted to evaluate the association between SIRI and stroke. RCS were employed to explore the dose–response relationship between SIRI and stroke. To assess the moderating effect of sex, the strength of the associations between hypertension, stroke, and SIRI was measured separately for males and females. In addition, to assess the predictive performance of hypertension and SIRI for stroke, we conducted Receiver Operating Characteristic (ROC) curve analysis using the fully adjusted multivariate logistic regression model (Model 3). This analysis provided key metrics, including the Area Under the Curve (AUC), sensitivity, specificity, positive predictive value (PPV), and negative predictive value (NPV), to evaluate the model’s discriminative power and predictive accuracy. The optimal classification threshold was established using the Youden index, defined as sensitivity plus specificity minus one.

To further elucidate the role of SIRI in the relationship between hypertension and stroke, mediation analysis was performed using the mediate function from the mediation package with 1,000 bootstrap samples. This estimated the average causal mediation effect (ACME), direct effect (ADE), total effect, and proportion mediated. Statistical significance was determined based on bootstrap-derived confidence intervals (CIs) and *p*-values. The use of bootstrap resampling enhanced the robustness of the mediation estimates. Additionally, mediation analysis was conducted separately for sex subgroups.

## 3 Results

### 3.1 Participant characteristics

In this study, a total of 9,699 adults aged 20 years or older were included. Among the participants, 70% were female, 30% were male, and 424 individuals (4.37%) had a history of stroke. Table 1 summarizes the demographic and clinical characteristics of participants stratified by stroke status. Significant differences were observed between stroke and non-stroke participants in terms of age, sex, marital status, race/ethnicity, educational attainment, PIR, smoking status, coronary heart disease, cancer, diabetes, hypertension, and SIRI (*p* < 0.05). Specifically, stroke patients were more likely to be older (≥ 60 years: 76% vs. 32%), female (77% vs. 70%), living alone (51% vs. 36%), and predominantly non-Hispanic White (71% vs. 60%) or non-Hispanic Black (16% vs. 15%). Stroke patients also exhibited lower levels of educational attainment, with 33% not completing high school, and higher prevalence rates of smoking (37% vs. 25%), hypertension (84% vs. 50%), coronary heart disease (18% vs. 3.4%), diabetes (36% vs. 15%), and cancer (23% vs. 9.8%). Regarding inflammatory markers, the SIRI levels were significantly higher in stroke patients compared to non-stroke participants [median (interquartile range): 1.17 (0.76, 1.80) vs. 1.02 (0.69, 1.47)]. However, no significant differences were found in BMI, alcohol consumption, or dyslipidemia (*p* > 0.05).

**Table 1:** Participant characteristics categorized by stroke status.

Characteristic	Overall, *N* = 9,699	Non-stroke, *N* = 9,275	Stroke, *N* = 424	*p*-value
Age (%)				<0.001
20–39 years	2,860 (32%)	2,851 (33%)	9 (3.0%)	
40–59 years	2,878 (35%)	2,812 (35%)	66 (21%)	
60 years and older	3,961 (33%)	3,612 (32%)	349 (76%)	
Sex (%)				0.015
Female	6,897 (70%)	6,593 (70%)	304 (77%)	
Male	2,802 (30%)	2,682 (30%)	120 (23%)	
Marital status (%)				<0.001
Widowed/divorced/separated/never married	3,945 (37%)	3,717 (36%)	228 (51%)	
Married/living with partner	5,754 (63%)	5,558 (64%)	196 (49%)	
Race (%)				<0.001
Mexican American	1,802 (9.2%)	1,759 (9.4%)	43 (3.7%)	
Other Hispanic	868 (6.1%)	838 (6.2%)	30 (3.4%)	
Non-Hispanic White	3,648 (60%)	3,432 (60%)	216 (71%)	
Non-Hispanic Black	2,323 (15%)	2,214 (15%)	109 (16%)	
Other Race (Including Multi-Racial)	1,058 (9.3%)	1,032 (9.4%)	26 (5.9%)	
Educational attainment (%)				<0.001
Less than high school	3,034 (22%)	2,874 (22%)	160 (33%)	
High school graduate	2,291 (25%)	2,188 (25%)	103 (26%)	
More than high school	4,374 (52%)	4,213 (53%)	161 (41%)	
PIR (%)				<0.001
≤1	2,392 (19%)	2,289 (18%)	103 (22%)	
1 to ≤3	4,404 (42%)	4,179 (41%)	225 (53%)	
>3	2,903 (40%)	2,807 (40%)	96 (26%)	
BMI (%)				0.6
<25 kg/m^2^	2,668 (28%)	2,554 (29%)	114 (26%)	
25 to <30 kg/m^2^	3,060 (31%)	2,928 (31%)	132 (33%)	
≥30 kg/m^2^	3,971 (41%)	3,793 (41%)	178 (41%)	
Smoking status (%)				<0.001
<100 cigarettes/lifetime	7,202 (74%)	6,946 (75%)	256 (63%)	
≥100 cigarettes/lifetime	2,497 (26%)	2,329 (25%)	168 (37%)	
Alcohol consumption (%)				0.4
<12 drinks/lifetime	4,801 (47%)	4,594 (47%)	207 (50%)	
≥12 drinks/lifetime	4,898 (53%)	4,681 (53%)	217 (50%)	
Dyslipidemia (%)				0.6
No	8,174 (83%)	7,834 (83%)	340 (82%)	
Yes	1,525 (17%)	1,441 (17%)	84 (18%)	
Coronary heart disease (%)				<0.001
No	9,273 (96%)	8,927 (97%)	346 (82%)	
Yes	426 (3.9%)	348 (3.4%)	78 (18%)	
Cancer (%)				<0.001
No	8,758 (90%)	8,425 (90%)	333 (77%)	
Yes	941 (10%)	850 (9.8%)	91 (23%)	
Diabetes (%)				<0.001
No	7,790 (84%)	7,531 (85%)	259 (64%)	
Yes	1,909 (16%)	1,744 (15%)	165 (36%)	
Hypertension (%)				<0.001
No	4,369 (48%)	4,308 (50%)	61 (16%)	
Yes	5,330 (52%)	4,967 (50%)	363 (84%)	
SIRI (×10^3^ cells/μL)	1.02 (0.69, 1.48)	1.02 (0.69, 1.47)	1.17 (0.76, 1.80)	<0.001

Median (25, 75%) for continuous variables: the p value was calculated by the Wilcoxon rank-sum test; (%) for categorical variables: the *p* value was calculated by the weighted Chi-square test. BMI, body mass index; PIR, poverty-income ratio; SIRI, systemic inflammation response index.

Stratified analyses were further performed according to hypertension status and sex. Supplementary Table 1 outlines the characteristics of participants grouped by hypertension status. The hypertensive group was notably older, with 51% of individuals aged ≥60 years, in contrast to 14% in the non-hypertensive group. The SIRI in the hypertensive group was elevated at 1.07 (0.74–1.58), compared to 0.97 (0.65–1.38) in the non-hypertensive group. Additionally, the hypertensive group exhibited significantly higher prevalence rates of stroke (6.1% vs. 1.2%), coronary heart disease (6.3% vs. 1.4%), and diabetes (25% vs. 6.6%). Supplementary Table 2 details the characteristics stratified by sex. Hypertension prevalence was comparable between males and females (52% vs. 52%, *p* > 0.9). However, males showed a higher median SIRI of 1.08 (0.76–1.58) compared to 0.99 (0.67–1.44) in females (*p* < 0.001), alongside an increased burden of dyslipidemia (33% vs. 11%, *p* < 0.001) and coronary heart disease (5.1% vs. 3.4%, *p* = 0.002).

### 3.2 Univariate regression analysis of the stroke group and the non-stroke group

Univariate analysis identified significant associations between stroke risk and multiple factors (Supplementary Table 3). Age emerged as a critical determinant, with individuals aged 40–59 years (OR = 6.47, 95% CI = 2.72–15.4, *p* < 0.001) and those ≥60 years (OR = 26.7, 95% CI = 11.9–60.0, p < 0.001) exhibiting substantially higher odds of stroke compared to the 20–39 years reference group. Gender also influenced risk, with males demonstrating lower odds than females (OR = 0.690, 95% CI = 0.511–0.931, *p* = 0.0158). Hypertension and an elevated SIRI were prominent risk factors (OR = 5.29, 95% CI = 3.76–7.43, p < 0.001; OR = 1.22, 95% CI = 1.12–1.32, p < 0.001, respectively). Additional factors, including marital status, education level, race, PIR, coronary heart disease, cancer, and diabetes, were also linked to stroke risk. BMI (31, 32), dyslipidemia (33, 34), and alcohol consumption (35, 36) were also included in subsequent multivariate analyses due to their well-documented links to stroke in existing literature, although they did not achieve statistical significance in the univariate analysis (*p* > 0.05).

### 3.3 Association between hypertension and stroke

Multivariate logistic regression analysis revealed a robust association between hypertension and stroke, with a fully adjusted odds ratio (OR) of 2.06 (Model 3; 95% CI = 1.42–2.99, *p* < 0.001) after accounting for demographic and clinical covariates (Table 2). The effect size decreased progressively from an unadjusted OR of 5.29 (Model 1; 95% CI = 3.76–7.43, *p* < 0.001) to 2.20 following adjustments for demographic factors alone (Model 2; 95% CI = 1.56–3.10, *p* < 0.001). Sex-stratified analyses demonstrated significant associations in both males and females. Specifically, the adjusted OR was higher in males (Model 3: OR = 2.41, 95% CI = 1.31–4.43, *p* = 0.005) compared to females (Model 3: OR = 1.97, 95% CI = 1.25–3.11, *p* = 0.004). Unadjusted associations were notably stronger in both sexes (males: OR = 5.11, 95% CI = 2.98–8.77, *p* < 0.001; females: OR = 5.35, 95% CI = 3.52–8.12, *p* < 0.001). To minimize potential multicollinearity, sex-specific models excluded adjustment for sex. Notably, all associations remained statistically significant across sequential adjustments for confounding variables.

**Table 2:** The multivariate logistic regression analysis of stroke across hypertension.

Hypertension	Model 1		Model 2		Model 3	
	OR (95% CI)	*p*-value	OR (95% CI)	*p*-value	OR (95% CI)	*p*-value
Total	5.29 (3.76,7.43)	< 0.001	2.20 (1.56,3.10)	< 0.001	2.06 (1.42,2.99)	< 0.001
Male	5.11 (2.98,8.77)	< 0.001	2.50 (1.39,4.51)	0.003	2.41(1.31, 4.43)	0.005
Female	5.35 (3.52,8.12)	< 0.001	2.15 (1.41,3.26)	< 0.001	1.97 (1.25,3.11)	0.004

Model 1: Unadjusted. Model 2: adjusted for age, sex, marital status, race, education level, and PIR. Model 3: adjusted for age, sex, marital status, race, education level, PIR, BMI, smoking status, alcohol consumption, diabetes, hyperlipidemia, coronary heart disease, and cancer. In the gender subgroup, model 2 and model 3 did not adjust sex. 95% CI, 95% confidence interval; OR: Odds ratios; SIRI, systemic inflammation response index; BMI, body mass index; PIR, poverty-income ratio.

### 3.4 Association between hypertension and SIRI

Multivariate linear regression analyses revealed a significant association between hypertension and elevated SIRI levels (Model 3: *β* = 1.07, 95% CI = 1.03–1.12, *p* = 0.002) after accounting for demographic and clinical confounders (Table 3). The effect size displayed progressive reduction with sequential models, decreasing from an unadjusted *β* of 1.15 (Model 1; 95% CI = 1.11–1.20, *p* < 0.001) to 1.11 (Model 2; 95% CI = 1.07–1.16, *p* < 0.001) when adjusting for demographic variables. Sex-stratified analysis indicated divergent patterns: the more marked unadjusted association was seen in males (Model 1: β = 1.26, 95% CI = 1.16–1.36, *p* < 0.001), which attenuated to 1.10 (Model 3; 95% CI = 1.00–1.22, *p* = 0.046) in the fully adjusted model, while females experienced a more gradual reduction from β = 1.11 (Model 1; 95% CI = 1.06–1.16, *p* < 0.001) to β = 1.07 (Model 3; 95% CI = 1.02–1.13, *p* = 0.010). To avoid statistical collinearity, sex adjustment was omitted in stratified models by design. Notably, all the associations were still statistically significant (*p* < 0.05) throughout the iterative model building. While the lower confidence limit in men for model 3 (1.00) was close to the null level, indicating variability in clinical importance.

**Table 3:** The multivariate linear regression analysis of SIRI across hypertension.

SIRI	Model 1		Model 2		Model 3	
	β (95% CI)	*p*-value	*β* (95% CI)	*p*-value	*β* (95% CI)	*p*-value
Total	1.15 (1.11,1.20)	< 0.001	1.11 (1.07,1.16)	< 0.001	1.15 (1.11,1.20)	< 0.001
Male	1.26 (1.16,1.36)	< 0.001	1.14 (1.04,1.24)	0.004	1.26 (1.16,1.36)	< 0.001
Female	1.11 (1.06,1.16)	< 0.001	1.12 (1.06,1.18)	< 0.001	1.11 (1.06,1.16)	< 0.001

Model 1: Unadjusted. Model 2: adjusted for age, sex, marital status, race, education level, and PIR. Model 3: adjusted for age, sex, marital status, race, education level, PIR, BMI, smoking status, alcohol consumption, diabetes, hyperlipidemia, coronary heart disease, and cancer. In the sex subgroup, model 2 and model 3 did not adjust gender. 95% CI, 95% confidence interval; β: regression coefficient; SIRI, systemic inflammation response index; BMI, body mass index; PIR, poverty-income ratio.

### 3.5 Association between SIRI and stroke

**Figure 2:**
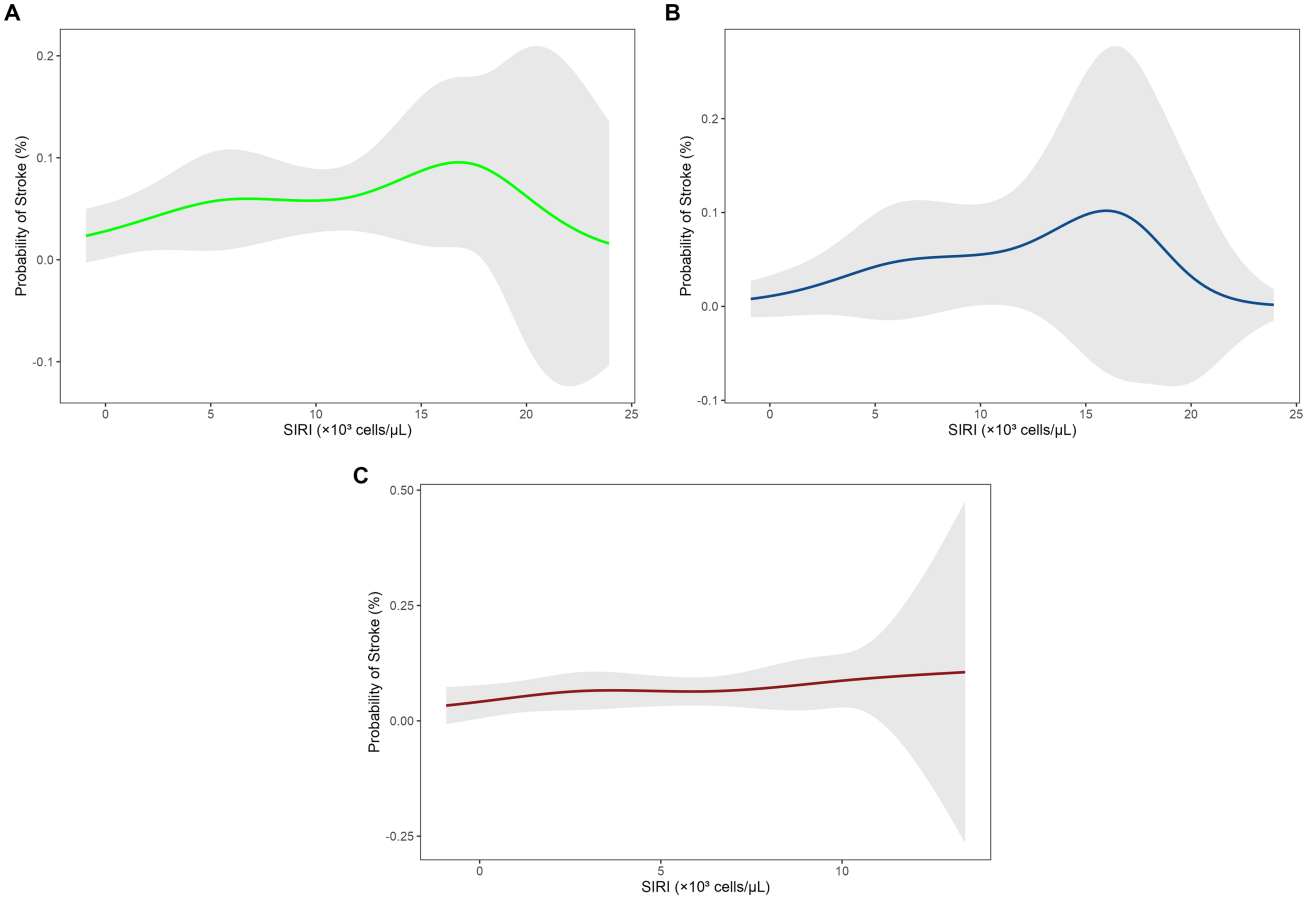
Restricted cubic spline (RCS) analysis of the relationship between SIRI levels and stroke risk. **(A)** Total population, **(B)** male group, **(C)** female group. SIRI, systemic inflammation response index; RCS, restricted cubic spline.

Increased SIRI levels were positively associated with the risk of stroke in a dose–response pattern in the fully adjusted model (Model 3: OR = 1.10, 95% CI = 1.00–1.21, *p* = 0.047) and this was supported by a significant trend (P for trend = 0.047). However, RCS analysis indicated nonlinear U-shaped correlation in the total population (Figure 2A), with stroke risk initially increasing with higher SIRI levels but decreasing at higher levels, contrary to the linear hypothesis.

Sex-stratified analyses presented divergent trends. In males, unadjusted associations (Model 1: OR = 1.25, 95% CI = 1.11–1.42, *p* < 0.001) attenuated to non-significance after adjustments for demographic and clinical factors (Model 3: OR = 1.12, 95% CI = 0.98–1.28, *p* = 0.11), with RCS curves indicating threshold effects at higher SIRI levels (Figure 2B; P for trend = 0.11). In females, transient significance was observed in models adjusted for demographic variables (Model 2: OR = 1.14, 95% CI = 1.01–1.30, *p* = 0.033), which vanished after further adjustment for clinical covariates (Model 3: OR = 1.07, 95% CI = 0.95–1.22, *p* = 0.30), with a relatively flat RCS trend (Figure 2C; P for trend = 0.25) (see Table 4).

**Table 4:** The multivariate logistic regression analysis and trend tests of stroke across SIRI.

SIRI	Model 1		Model 2		Model 3		
	OR (95% CI)	*p*-value	OR (95% CI)	*p*-value	OR (95% CI)	*p*-value	*p* for trend
Total	1.22 (1.12,1.32)	< 0.001	1.14 (1.04,1.25)	0.004	1.10 (1.00,1.21)	0.047	0.047026
Male	1.25 (1.11,1.42)	< 0.001	1.12 (0.98,1.27)	0.11	1.12 (0.98,1.28)	0.11	0.1094294
Female	1.22 (1.09,1.36)	< 0.001	1.14 (1.01,1.30)	0.033	1.07 (0.95,1.22)	0.3	0.2542953

Model 1: Unadjusted. Model 2: adjusted for age, sex, marital status, race, education level, and PIR. Model 3: adjusted for age, sex, marital status, race, education level, PIR, BMI, smoking status, alcohol consumption, diabetes, hyperlipidemia, coronary heart disease, and cancer. In the sex subgroup, model 2 and model 3 did not adjust sex. 95% CI, 95% confidence interval; OR: Odds ratios; SIRI, systemic inflammation response index; BMI, body mass index; PIR, poverty-income ratio.

### 3.6 Predictive performance of hypertension and SIRI for stroke

To assess the ability of hypertension and SIRI to predict stroke, we conducted ROC curve analyses (Supplementary Table 4). For hypertension, the AUC was 0.805 (95% CI: 0.788–0.823), with a sensitivity of 80.2%, specificity of 68.5%, PPV of 10.4%, and NPV of 98.7%. In sex-stratified analyses, males exhibited an AUC of 0.812 (95% CI: 0.783–0.841), with 81.7% sensitivity, 71.3% specificity, 11.3% PPV, and 98.9% NPV, whereas females showed an AUC of 0.808 (95% CI: 0.788–0.829), with 84.2% sensitivity, 64.4% specificity, 9.8% PPV, and 98.9% NPV. For SIRI, the AUC was 0.801 (95% CI: 0.783–0.819), with a sensitivity of 87.7%, specificity of 59.5%, PPV of 9.0%, and NPV of 99.1%. Among males, SIRI yielded an AUC of 0.809 (95% CI: 0.777–0.840), with 90.0% sensitivity, 61.7% specificity, 9.5% PPV, and 99.3% NPV, while females had an AUC of 0.804 (95% CI: 0.783–0.825), with 80.3% sensitivity, 66.4% specificity, 9.9% PPV, and 98.6% NPV. These findings indicate that both hypertension and SIRI demonstrate robust discriminatory capacity for stroke, with high NPVs underscoring their utility in ruling out stroke risk, particularly in males.

### 3.7 The mediating role of SIRI

**Figure 3:**
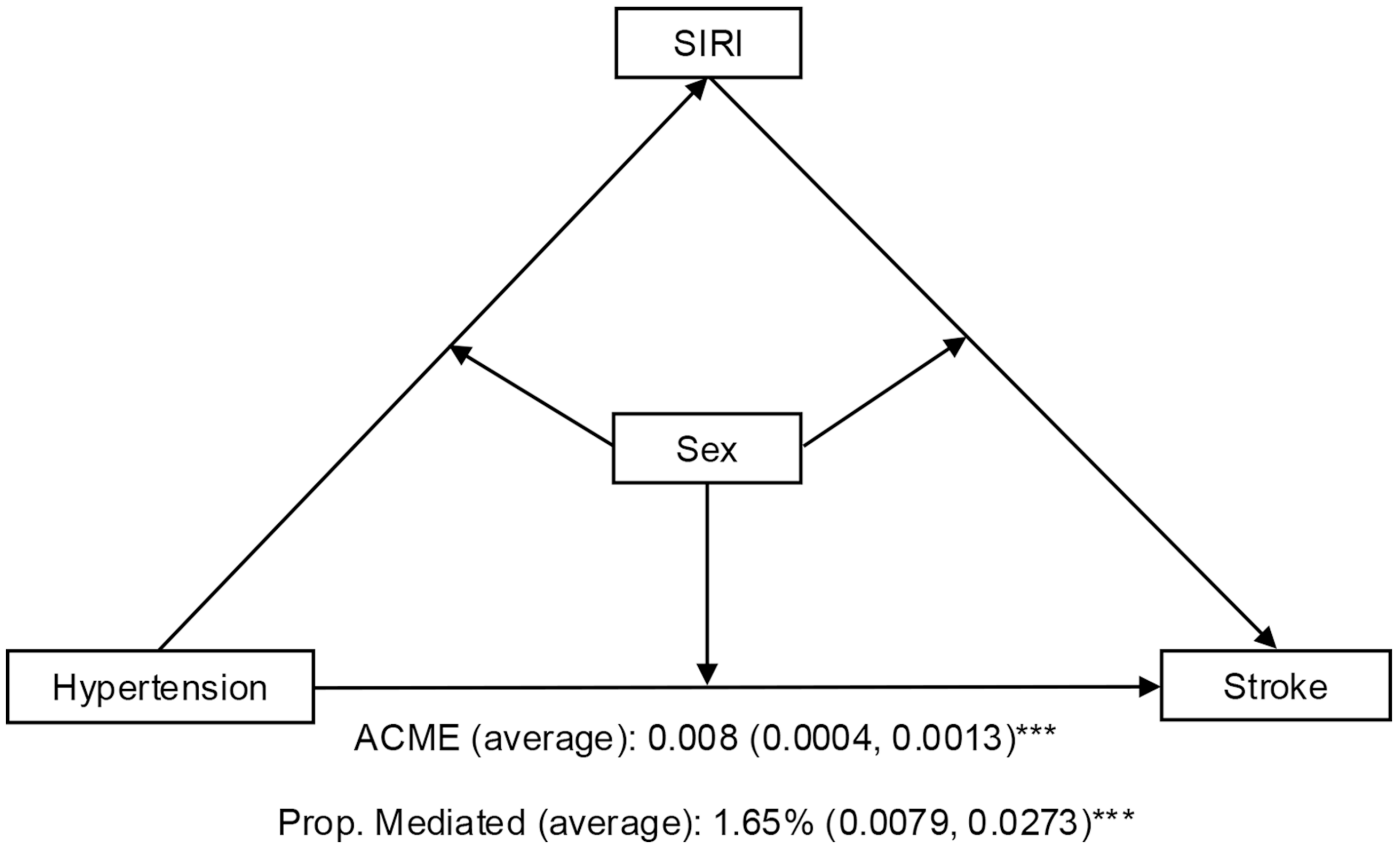
A path diagram of the mediation analysis of SIRI on hypertension and stroke, stratified by sex. ***p < 0.001. SIRI, systemic inflammation response index; ACEM, average causal mediation effect; Prop. Mediated, Proportion mediated.

SIRI partially mediated the association between hypertension and stroke, there were significant sex differences (Table 5, Figure 3). In the total population, hypertension had a direct effect on stroke (ADE = 0.0476, 95% CI = 0.040–0.0551, *p* < 2 × 10^−16^), while SIRI contributed a modest but statistically significant mediating effect (ACME = 0.0008, 95% CI = 0.0004–0.0013, *p* < 2 × 10^−16^), accounting for 1.65% (95% CI = 0.79–2.74%, *p* < 2 × 10^−16^) of the total effect. Sex-stratified analyses revealed stronger mediation in men than in women: SIRI mediated 3.38% of the total effect in men (ACME = 0.0013, 95% CI = 0.0005–0.0023, *p* = 0.002), but only 1.16% in women (ACME = 0.0006, 95% CI = 0.0002–0.0012, *p* = 0.01). Surprisingly, the direct effect of hypertension on stroke was more pronounced in women (ADE = 0.0526, 95% CI = 0.0420–0.0630, *p* < 2 × 10^−16^) than in men (ADE = 0.0366, 95% CI = 0.0248–0.0466, *p* < 2 × 10^−16^), suggesting sex-specific mechanisms for the hypertension-stroke relationship.

**Table 5:** SIRI mediating the association between hypertension and stroke.

SIRI	ACEM		ADE		Total effect		Proportion mediated	
	OR (95% CI)	*p* value	OR (95% CI)	*p* value	OR (95% CI)	*p* value	OR (95% CI)	*p* value
Total	0.000811 (0.000383, 0.001327)	<2 × 10^−16^	0.047579 (0.039718, 0.055133)	<2 × 10^−16^	0.048390 (0.040437, 0.056029)	<2 × 10^−16^	0.016519 (0.007899, 0.027377)	<2 × 10^−16^
Male	0.001280 (0.000491, 0.002278)	0.002	0.035557 (0.024796, 0.046585)	<2 × 10^−16^	0.036837 (0.025864, 0.048043)	<2 × 10^−16^	0.033811 (0.013590, 0.063429)	0.002
Female	0.000635 (0.000171, 0.001204)	0.01	0.052645 (0.042039, 0.063007)	<0.0001	0.053280 (0.042715, 0.063444)	<0.0001	0.011575 (0.003377, 0.023298)	0.01

95% CI, 95% confidence interval; OR: Odds ratios; SIRI, systemic inflammation response index; ACME, average causal mediation effect; ADE, average direct effect.

## 4 Discussion

While the established connection between hypertension and stroke is highly recognized, SIRI’s mediating effect in this association is yet to be widely researched. In this present study, we initially employed multivariate regression and RCS analyses to establish the correlations between hypertension, SIRI, and stroke, while ensuring that the conditions for mediation analysis were met. Additionally, ROC curve analyses confirmed the predictive utility of SIRI and hypertension for stroke, with superior performance. Despite the modest PPV, likely attributable to the low stroke prevalence in the study population, the high NPV affirmed their strong capacity to rule out stroke risk. Subsequently, we formally assessed the mediating effect of SIRI through sex-stratified causal mediation models. The results showed that SIRI partially mediates the risk of stroke from hypertension with higher effects observed among men compared to women. These findings provide critical insights for the development of targeted anti-inflammatory interventions aimed to reduce the burden of stroke in high-risk populations.

Hypertension is a firmly established independent risk factor for stroke which has been demonstrated by numerous comprehensive epidemiological studies (37–39). Our findings consisted with the current literature, having a high correlation between hypertension and higher risk of stroke. Notably, Walle-Hansen et al. (37) identified systolic blood pressure (SBP) ≥ 140 mmHg as a risk threshold for stroke, and Ren et al. (38) reported pulse pressure (PP) > 50 mmHg as a significant predictor in older populations. In addition to traditional hemodynamic parameters, Wang et al. further also uncovered the cumulative impact of elevated mean arterial pressure (MAP) on ischemic stroke occurrence (39). Interestingly, our sex-stratified analyses indicated a stronger association of hypertension with stroke in males than in females, a finding that was consistent with Wang et al.’s (39) cohort, wherein cumulative MAP had a steeper risk slope in men. These findings emphasize the importance of prioritizing hypertension screening and management in men, which could improve the accuracy and efficiency of stroke prevention and management strategies.

Hypertension plays a vital role in stroke pathogenesis through an intricate interrelated network of pathophysiological mechanisms, including oxidative stress-induced inflammatory interactions, cerebrovascular remodeling (eg, atherosclerosis and small-vessel disease), disruption of cerebral autoregulation, and imbalances within the autonomic nervous system. These mechanisms eventually culminate in ischemic or hemorrhagic stroke (40). Our findings particularly emphasized the inflammatory axis, showing that hypertension significantly increases SIRI levels, in line with earlier studies associating hypertension with inflammatory biomarkers (41–43). The following mechanisms may further elucidate the relationship between hypertension and systemic inflammation. First, the chronic mechanical stress inherent to hypertension could lead to the injury of vascular endothelial cells, resulting in endothelial dysfunction and the secretion of pro-inflammatory cytokines such as interleukin-6 (IL-6) and tumor necrosis factor-alpha (TNF-*α*), and chemokines like C-C motif chemokine ligand 2 (CCL2). These factors promote the recruitment of neutrophils and monocytes to the vascular wall (5, 6). Concurrently, activation of oxidative stress mechanisms, particularly NADPH oxidase, generates excess reactive oxygen species (ROS), which directly injure endothelial cells while also increasing the expression of pro-inflammatory genes through nuclear factor kappa-light-chain-enhancer of activated B cells (NF-κB) signaling, thereby developing a damage-inflammation loop (44, 45). Immune cell infiltration mediated by chemokines including Th1 and Th17 cells sustains chronic inflammation coupled with endothelial injury and oxidative stress (45, 46). Further, ROS promote vascular permeability, platelet aggregation, and release of coagulation factors, adding to a pro-thrombotic microenvironment that exacerbates local inflammation and elevates the risk of ischemic stroke (45, 47). All these processes hence drive systemic inflammation, as seen in elevated SIRI values, thereby enhancing stroke susceptibility (5, 6, 11).

Meanwhile, in the total population, our analyses revealed SIRI to be an independent predictor of stroke risk within fully adjusted models, consistent with existing evidence implicating chronic inflammation in cerebrovascular pathogenesis (15, 48, 49). The underlying mechanisms potentially involve multiple processes: Chronic inflammation may contribute to vascular endothelial damage and thrombosis by inducing oxidative stress, thereby disrupting cerebral blood flow (50, 51). Ischemia trigger immune cell activation and recruitment of immune cells in the brain tissue, including microglia and peripheral immune cells, into ischemic regions. These cells release pro-inflammatory mediators, such as TNF-*α* and interleukin-1β (IL-1β), which enhance further neuronal damage (52, 53). Excessive activation of the complement system initiates the expression of adhesion molecules, such as intercellular adhesion molecule 1 (ICAM-1) and vascular cell adhesion molecule 1 (VCAM-1), to promote neutrophil adherence and subsequent release of proteases and reactive oxygen species. This process injures vascular endothelial cells directly, increases blood–brain barrier (BBB) permeability, and facilitates the infiltration of immune cells, including monocytes and neutrophils, into the brain parenchyma, thereby facilitating stroke development (54). Additionally, chronic inflammation is at the core of all stages of atherosclerosis, increasing intraplaque lipid deposition, macrophage infiltration, and plaque rupture, thereby leading to thromboembolic complications (55).

Besides, even though trend tests indicated a significant linear relationship between SIRI and stroke risk, RCS curve analyses revealed a nonlinear U-shaped risk trajectory with stroke risk increasing but then plateaus or decreases at higher SIRI values. This shape of the RCS curve suggested a threshold effect wherein stroke risk escalates with inflammation to a point but then plateaus or decreases after reaching a specific SIRI threshold. Yet, in sex-stratified multivariate logistic regression analyses, the association between SIRI and stroke risk became non-significant in both women and men after control for all confounding variables. RCS curve analysis subsequently demonstrated that men experienced a more rapid rise in risk of stroke with increased levels of SIRI with a steeper slope than women. Conversely, women showed a blunted risk curve and a less pronounced overall curve, showing a sex-specific threshold effect whereby the risk of stroke in men is more sensitive to heightened inflammation. This male-predominant trend aligned with Chen et al. (56), where a high correlation was found between peripheral markers of inflammation, such as pan-immune inflammation value (PIV), and incidence of stroke in hypertensive populations. This may be elucidated by estrogen’s anti-inflammatory protective effect in women, decreasing stroke risk, whereas male behavior such as alcoholism and smoking directly contributes to increasing stroke risk (21, 57).

In the mediation analysis for our study, SIRI modestly mediated the relationship of hypertension with stroke to the level of 1.65% of the total effect. This suggested that hypertension may elevate stroke risk by impacting inflammatory processes. There is mounting evidence for the role of inflammation linked to hypertension in the pathogenesis of stroke. For instance, in a cohort study, Cai et al. (58) demonstrated that high SIRI levels were strongly associated with stroke risk and its subtypes in older hypertensive patients. Similarly, Jiménez et al. (14) showed relationships between hs-CRP, sICAM-1, hypertension status, and stroke risk in women. Furthermore, Huang et al. (59) observed that inflammatory markers such as SIRI and C-reactive protein (CRP) partially mediated the relationship between insulin resistance and stroke in hypertensive patients. Additionally, our research revealed that the mediation effect of SIRI was greater in men compared to women, which implied that males might exhibit greater inflammatory susceptibility in the hypertension-stroke continuum. These findings hold important clinical ramifications. SIRI-based risk stratification potentially enhances the earlier detection of patients at high risk of stroke and hence enables the use of tailored anti-inflammatory treatment in combination with conventional blood pressure management. Public health strategies should incorporate sex-specific approaches, emphasizing inflammatory biomarker screening in hypertensive men and the targeting of non-inflammatory mechanisms in women. Nevertheless, the limited mediation proportion underscores the multifactorial nature of stroke pathogenesis and necessitates probing synergistic biomarkers to maximize predictive power of risk models.

Our research had several limitations. Firstly, the cross-sectional design did not allow us to establish a causal link between hypertension, SIRI, and stroke. Secondly, although the NHANES dataset reflects the U. S. population, it depends on self-reported stroke information, which may result in underreporting, and it lacks details about the use of anti-inflammatory medications. Furthermore, despite accounting for various confounding factors, the influence of unmeasured variables might still affect our findings. A key point to highlight is the dataset’s omission of stroke subtype information and its inclusion of only a limited number of very elderly individuals. This restricts our ability to investigate SIRI’s role across distinct stroke categories, such as lacunar and non-lacunar infarctions, which differ substantially in their underlying mechanisms and outcomes or within vulnerable groups like the very elderly, whose risk characteristics are unique (60, 61). Moving forward, longitudinal studies incorporating detailed data on stroke subtypes and age and sex-specific traits are necessary to confirm causality and examine SIRI’s potential as a predictor across diverse populations.

## 5 Conclusion

In summary, the research revealed that SIRI may mediate some of the association between hypertension and stroke, with the effect being stronger in males.

## Data Availability

The data analyzed in this study were obtained from the National Health and Nutrition Examination Survey (NHANES) 2001–2016, publicly available at: https://wwwn.cdc.gov/nchs/nhanes/default.aspx. The original contributions and derived analyses presented in the article/Supplementary material can be accessed, and further inquiries can be directed to the corresponding authors.

## Glossary

ACME: Average Causal Mediation Effect
ADE: Average Direct Effect
AHA/ACC: American Heart Association/American College of Cardiology
AUC: Area Under the Curve
BBB: Blood–Brain Barrier
BMI: Body Mass Index
CCL2: C-C Motif Chemokine Ligand 2
CBC: Complete Blood Count
CDC: Centers for Disease Control and Prevention
CI: Confidence Interval
CRP: C-Reactive Protein
DALYs: Disability-Adjusted Life Years
DBP: Diastolic Blood Pressure
GBD: Global Burden of Disease
HbA1c: Glycated Hemoglobin
HDL: High-Density Lipoprotein
hs-CRP: High-Sensitivity C-Reactive Protein
ICAM-1: Intercellular Adhesion Molecule 1
IL-1β: Interleukin-1 Beta; IL-6: Interleukin-6
LDL: Low-Density Lipoprotein
MAP: Mean Arterial Pressure
MEC: Mobile Examination Centers
NCHS: National Center for Health Statistics
NF-κB: Nuclear Factor Kappa-Light-Chain-Enhancer of Activated B Cells
NHANES: National Health and Nutrition Examination Survey
NPV: Negative Predictive Value
NSTEMI: Non-ST-Elevation Myocardial Infarction
OR: Odds Ratio
PIR: Poverty-Income Ratio
PIV: Pan-Immune Inflammation Value
PP: Pulse Pressure
PPV: Positive Predictive Value
ROC: Receiver Operating Characteristic
RCS: Restricted Cubic Splines
ROS: Reactive Oxygen Species
SBP: Systolic Blood Pressure
SIRI: Systemic Inflammation Response Index
TC: Total Cholesterol
TG: Triglycerides
Th1: T-Helper 1 Cell
Th17: T-Helper 17 Cell
TNF-*α*: Tumor Necrosis Factor-Alpha
VCAM-1: Vascular Cell Adhesion Molecule 1
WHO: World Health Organization
