# Supplementary material for: Systemic inflammatory response index mediates the association between stroke and hypertension: a cross-sectional study from NHANES 2001 to 2016
Source: Front Neurol. 2025 Jul 30;16:1603241. doi: 10.3389/fneur.2025.1603241 (PMC12351121; doi:10.3389/fneur.2025.1603241)
Supplement: Supplementary file 1 [file Table_1.docx]

Supplementary Material

## Supplementary Tables

**Supplementary Table 1** Participant characteristics categorized by hypertension status

| Characteristic | Overall,  N = 9699 | Non-hypertension,  N = 4369 | Hypertension,  N = 5330 | p-value |
| --- | --- | --- | --- | --- |
| Age (%) |  |  |  | <0.001 |
| 20-39 years | 2,860 (32%) | 2,268 (52%) | 592 (13%) |  |
| 40-59 years | 2,878 (35%) | 1,331 (34%) | 1,547 (36%) |  |
| 60 years and older | 3,961 (33%) | 770 (14%) | 3,191 (51%) |  |
| Sex (%) |  |  |  | >0.9 |
| Female | 6,897 (70%) | 3,149 (70%) | 3,748 (70%) |  |
| Male | 2,802 (30%) | 1,220 (30%) | 1,582 (30%) |  |
| Marital status (%) |  |  |  | <0.001 |
| Widowed/divorced/  separated/never married | 3,945 (37%) | 1,587 (34%) | 2,358 (39%) |  |
| Married/ Living with partner | 5,754 (63%) | 2,782 (66%) | 2,972 (61%) |  |
| Race (%) |  |  |  | <0.001 |
| Mexican American | 1,802 (9.2%) | 984 (12%) | 818 (6.6%) |  |
| Other Hispanic | 868 (6.1%) | 422 (7.3%) | 446 (5.0%) |  |
| Non-Hispanic White | 3,648 (60%) | 1,491 (56%) | 2,157 (64%) |  |
| Non-Hispanic Black | 2,323 (15%) | 901 (14%) | 1,422 (16%) |  |
| Other Race  (Including multi-racial) | 1,058 (9.3%) | 571 (11%) | 487 (8.0%) |  |
| Educational attainment (%) |  |  |  | <0.001 |
| Less than high school | 3,034 (22%) | 1,213 (20%) | 1,821 (25%) |  |
| High school graduate | 2,291 (25%) | 954 (23%) | 1,337 (28%) |  |
| More than high school | 4,374 (52%) | 2,202 (57%) | 2,172 (48%) |  |
| PIR (%) |  |  |  | <0.001 |
| ≤1 | 2,392 (19%) | 1,159 (21%) | 1,233 (17%) |  |
| 1 to ≤3 | 4,404 (42%) | 1,854 (39%) | 2,550 (45%) |  |
| >3 | 2,903 (40%) | 1,356 (40%) | 1,547 (39%) |  |
| BMI (%) |  |  |  | <0.001 |
| <25 kg/m² | 2,668 (28%) | 1,567 (37%) | 1,101 (20%) |  |
| 25 to <30 kg/m² | 3,060 (31%) | 1,412 (31%) | 1,648 (30%) |  |
| ≥30 kg/m² | 3,971 (41%) | 1,390 (32%) | 2,581 (50%) |  |
| Smoking status (%) |  |  |  | <0.001 |
| <100 cigarettes/lifetime | 7,202 (74%) | 3,409 (77%) | 3,793 (71%) |  |
| ≥100 cigarettes/lifetime | 2,497 (26%) | 960 (23%) | 1,537 (29%) |  |
| Alcohol consumption (%) |  |  |  | 0.004 |
| <12 drinks/lifetime   ≥12 drinks/lifetime | 4,801 (47%)  4,898 (53%) | 2,213 (50%)  2,156 (50%) | 2,588 (45%)  2,742 (55%) |  |
| Dyslipidemia (%) |  |  |  | 0.019 |
| No | 8,174 (83%) | 3,740 (84%) | 4,434 (82%) |  |
| Yes | 1,525 (17%) | 629 (16%) | 896 (18%) |  |
| Coronary heart disease (%) |  |  |  | <0.001 |
| No | 9,273 (96%) | 4,309 (99%) | 4,964 (94%) |  |
| Yes | 426 (3.9%) | 60 (1.4%) | 366 (6.3%) |  |
| Cancer (%) |  |  |  | <0.001 |
| No | 8,758 (90%) | 4,121 (94%) | 4,637 (86%) |  |
| Yes | 941 (10%) | 248 (6.4%) | 693 (14%) |  |
| Diabetes (%) |  |  |  | <0.001 |
| No | 7,790 (84%) | 4,013 (93%) | 3,777 (75%) |  |
| Yes | 1,909 (16%) | 356 (6.6%) | 1,553 (25%) |  |
| Stroke (%) |  |  |  | <0.001 |
| No | 9,275 (96%) | 4,308 (99%) | 4,967 (94%) |  |
| Yes | 424 (3.7%) | 61 (1.2%) | 363 (6.1%) |  |
| SIRI (×10³ cells/μL) | 1.02  (0.69, 1.48) | 0.97  (0.65, 1.38) | 1.07  (0.74, 1.58) | <0.001 |

Notes: Median (25%, 75%) for continuous variables: the P value was calculated by the Wilcoxon rank-sum test; (%) for categorical variables: the P value was calculated by the weighted Chi-square test.

Abbreviations: BMI, body mass index; PIR, poverty-income ratio; SIRI, systemic inflammation response index.

**Supplementary Table 2** Participant characteristics categorized by sex

| Characteristic | Overall,  N = 9699 | Female,  N = 6897 | Male,  N = 2802 | p-value |
| --- | --- | --- | --- | --- |
| Age (%) |  |  |  | <0.001 |
| 20-39 years | 2,860 (32%) | 1,997 (30%) | 863 (36%) |  |
| 40-59 years | 2,878 (35%) | 2,053 (34%) | 825 (36%) |  |
| 60 years and older | 3,961 (33%) | 2,847 (36%) | 1,114 (28%) |  |
| Marital status (%) |  |  |  | <0.001 |
| Widowed/divorced/  separated/never married | 3,945 (37%) | 3,047 (40%) | 898 (30%) |  |
| Married/ Living with partner | 5,754 (63%) | 3,850 (60%) | 1,904 (70%) |  |
| Race (%) |  |  |  | <0.001 |
| Mexican American | 1,802 (9.2%) | 1,438 (10%) | 364 (7.2%) |  |
| Other Hispanic | 868 (6.1%) | 663 (6.5%) | 205 (5.2%) |  |
| Non-Hispanic White | 3,648 (60%) | 2,502 (59%) | 1,146 (62%) |  |
| Non-Hispanic Black | 2,323 (15%) | 1,606 (15%) | 717 (15%) |  |
| Other Race  (Including multi-racial) | 1,058 (9.3%) | 688 (8.6%) | 370 (11%) |  |
| Educational attainment (%) |  |  |  | 0.012 |
| Less than high school | 3,034 (22%) | 2,235 (23%) | 799 (20%) |  |
| High school graduate | 2,291 (25%) | 1,603 (26%) | 688 (25%) |  |
| More than high school | 4,374 (52%) | 3,059 (51%) | 1,315 (55%) |  |
| PIR (%) |  |  |  | <0.001 |
| ≤1 | 2,392 (19%) | 1,790 (19%) | 602 (16%) |  |
| 1 to ≤3 | 4,404 (42%) | 3,146 (43%) | 1,258 (40%) |  |
| >3 | 2,903 (40%) | 1,961 (38%) | 942 (44%) |  |
| BMI (%) |  |  |  | <0.001 |
| <25 kg/m² | 2,668 (28%) | 1,838 (29%) | 830 (28%) |  |
| 25 to <30 kg/m² | 3,060 (31%) | 2,067 (29%) | 993 (35%) |  |
| ≥30 kg/m² | 3,971 (41%) | 2,992 (42%) | 979 (37%) |  |
| Smoking status (%) |  |  |  | <0.001 |
| <100 cigarettes/lifetime | 7,202 (74%) | 5,368 (76%) | 1,834 (69%) |  |
| ≥100 cigarettes/lifetime | 2,497 (26%) | 1,529 (24%) | 968 (31%) |  |
| Alcohol consumption (%) |  |  |  | 0.4 |
| <12 drinks/lifetime   ≥12 drinks/lifetime | 4,801 (47%)  4,898 (53%) | 5,368 (76%)  1,529 (24%) | 1,281 (46%)  1,521 (54%) |  |
| Hypertension (%) |  |  |  | >0.9 |
| No | 4,369 (48%) | 3,149 (48%) | 1,220 (48%) |  |
| Yes | 5,330 (52%) | 3,748 (52%) | 1,582 (52%) |  |
| Dyslipidemia (%) |  |  |  | <0.001 |
| No | 8,174 (83%) | 6,177 (89%) | 1,997 (67%) |  |
| Yes | 1,525 (17%) | 720 (11%) | 805 (33%) |  |
| Coronary heart disease (%) |  |  |  | 0.002 |
| No | 9,273 (96%) | 6,654 (97%) | 2,619 (95%) |  |
| Yes | 426 (3.9%) | 243 (3.4%) | 183 (5.1%) |  |
| Cancer (%) |  |  |  | 0.003 |
| No | 8,758 (90%) | 6,228 (89%) | 2,530 (91%) |  |
| Yes | 941 (10%) | 669 (11%) | 272 (8.6%) |  |
| Diabetes (%) |  |  |  | 0.7 |
| No | 7,790 (84%) | 5,562 (84%) | 2,228 (84%) |  |
| Yes | 1,909 (16%) | 1,335 (16%) | 574 (16%) |  |
| Stroke (%) |  |  |  | 0.015 |
| No | 9,275 (96%) | 6,593 (96%) | 2,682 (97%) |  |
| Yes | 424 (3.7%) | 304 (4.1%) | 120 (2.8%) |  |
| SIRI (×10³ cells/μL) | 1.02  (0.69, 1.48) | 0.99  (0.67, 1.44) | 1.08  (0.76, 1.58) | <0.001 |

Notes: Median (25%, 75%) for continuous variables: the P value was calculated by the Wilcoxon rank-sum test; (%) for categorical variables: the P value was calculated by the weighted Chi-square test.

Abbreviations: BMI, body mass index; PIR, poverty-income ratio; SIRI, systemic inflammation response index.

**Supplementary Table 3** Univariate regression analysis of the stroke group and the non-stroke group

| Characteristic | Odds ratio (95% CI) | p-value |
| --- | --- | --- |
| Age (%) |  |  |
| 20-39 years | Reference |  |
| 40-59 years | 6.47 (2.72, 15.4) | <0.001 |
| 60 years and older | 26.7 (11.9, 60.0) | <0.001 |
| Male (%) | 0.690 (0.511, 0.931) | 0.0158 |
| Marital status (%) | 0.547 (0.421, 0.709) | <0.001 |
| Race (%) |  |  |
| Mexican American | Reference |  |
| Other Hispanic | 1.39 (0.736, 2.62) | 0.308 |
| Non-Hispanic White | 2.99 (1.99, 4.48) | <0.001 |
| Non-Hispanic Black | 2.68 (1.80, 4.00) | <0.001 |
| Other Race (Including Multi-Racial) | 1.58 (0.858, 2.90) | 0.141 |
| Educational attainment (%) |  |  |
| Less than high school | Reference |  |
| High school graduate | 0.678 (0.509, 0.903) | 0.008 |
| More than high school | 0.520 (0.397, 0.682) | <0.001 |
| PIR (%) |  |  |
| ≤1 | Reference |  |
| 1 to ≤3 | 1.07 (0.786, 1.45) | 0.669 |
| >3 | 0.538 (0.379, 0.766) | <0.001 |
| BMI (%) |  |  |
| <25 kg/m² | Reference |  |
| 25 to <30 kg/m² | 1.17 (0.844, 1.64) | 0.337 |
| ≥30 kg/m² | 1.08 (0.824, 1.42) | 0.564 |
| Smoking status (%) | 1.75 (1.35, 2.29) | <0.001 |
| Alcohol consumption (%) | 0.908 (0.720, 1.14) | 0.410 |
| Dyslipidemia (%) | 1.08 (0.787, 1.47) | 0.645 |
| Coronary heart disease (%) | 6.46 (4.61, 9.05) | <0.001 |
| Cancer (%) | 2.77 (2.04, 3.76) | <0.001 |
| Diabetes (%) | 3.18 (2.44, 4.16) | <0.001 |
| Hypertension (%) | 5.29 (3.76, 7.43) | <0.001 |
| SIRI (×10³ cells/μL) | 1.22 (1.12, 1.32) | <0.001 |

Abbreviations: BMI, body mass index; PIR, poverty-income ratio; SIRI, systemic inflammation response index.

**Supplementary Table 4** Subgroup analysis of the stroke predictive ability of hypertension and SIRI

| Characteristic | Threshold | Sensitivity (%) | Specificity (%) | PPV (%) | NPV (%) | AUC（95% CI） |
| --- | --- | --- | --- | --- | --- | --- |
| Hypertension | 0.045 | 0.802 | 0.685 | 0.104 | 0.987 | 0.805 (0.788-0.823) |
| Male | 0.041 | 0.817 | 0.713 | 0.113 | 0.989 | 0.812 (0.783-0.841) |
| Female | 0.040 | 0.842 | 0.644 | 0.098 | 0.989 | 0.808 (0.788-0.829) |
| SIRI | 0.033 | 0.877 | 0.595 | 0.090 | 0.991 | 0.801 (0.783-0.819) |
| Male | 0.027 | 0.900 | 0.617 | 0.095 | 0.993 | 0.809 (0.777-0.840) |
| Female | 0.045 | 0.803 | 0.664 | 0.099 | 0.986 | 0.804 (0.783-0.825) |

Abbreviations: SIRI, systemic inflammation response index; PPV, positive predictive value; NPV, negative predictive value; AUC, area under the curve.
